# The first record of Telemidae from Kenya, with the description of two new species (Arachnida, Araneae)

**DOI:** 10.3897/zookeys.725.15059

**Published:** 2017-12-29

**Authors:** Yang Song, Huifeng Zhao, Yufa Luo, Grace M. Kioko, Esther N. Kioko, Shuqiang Li

**Affiliations:** 1 School of Life and Environment Sciences, Gannan Normal University, Ganzhou, Jiangxi 341000, China; 2 Institute of Zoology, Chinese Academy of Sciences, Beijing 100101, China; 3 Zoology Department, National Museums of Kenya, Box 40658-00100, Nairobi, Kenya; 4 University of the Chinese Academy of Sciences, Beijing 100049, China

**Keywords:** East Africa, Haplogynae, new record, taxonomy

## Abstract

Two new species of Telemidae from Kenya are described: *Guhua kakamegaensis* Zhao & Li, **gen. et sp. n.**, *Apneumonella taitatavetaensis* Zhao & Li, **sp. n.** Holotypes are deposited in the National Museum of Kenya in Nairobi, and all paratypes are deposited in the Institute of Zoology, Chinese Academy of Sciences in Beijing. A distribution map of Telemidae found in Kenya is presented.

## Introduction

Telemidae Fage, 1913 is a relatively small family consisting of nine genera and 69 species (World Spider Catalog 2017), which are unevenly distributed in rainforest and karst regions of tropical Africa, Europe, East and Southeast Asia and the New World. The majority of the species are reported to occur in Southwest China and Southeast Asia, especially *Telema* Simon, 1882 and *Pinelema* Wang & Li, 2012, but 20 species of this family are fragmentally distributed in other regions of the world. There are three known genera of Telemidae in Africa: *Apneumonella* Fage, 1921, *Cangoderces* Harington, 1951 and *Seychellia* Saaristo, 1978. *Apneumonella* is composed of two species, the type species is from Tanzania (Fage 1921), and the other described species is found in Sumatra, Indonesia (Brignoli 1977). All five species of *Cangoderces* were discovered in western and southern African countries: Cameroon, Ivory Coast and South Africa (Harington 1951, Baert 1985, Wang and Li 2011). The genus *Seychellia* is composed of five species, which occur in Seychelles, Cameroon, Ivory Coast and China (Saaristo 1978, Brignoli 1980, Baert 1985, Lin and Li 2008, Wang and Li 2011). In this paper, we present the first report of Telemidae in Kenya. The new monotypic genus *Guhua* gen. n. is established based on new well-defined morphological characters.

## Materials and methods

All specimens were examined and measured using a Leica M205 C stereomicroscope. The bodies, male palps, and receptacles were photographed using an Olympus C7070 digital camera mounted on an Olympus SZX12 stereomicroscope. Images were combined using Helicon Focus version 6.7.1 image stacking software (http://www.heliconsoft.com). Further morphological details were observed under an Olympus BX41 compound light microscope. The left palps of the male were photographed with a Hitachi SU8010 Scanning Electron Microscope. Female genitalia were removed and treated in lactic acid before being photographed. All measurements are reported in millimeters. Leg measurements are shown as: total length (femur, patella, tibia, metatarsus, and tarsus). The following abbreviations are used in the figures:

**Em** embolus;

**Re** receptacle;

**Rs** receptacle scape;

**SO** secretory orifice;

**Ta** tegular apophysis.

Type specimens were deposited in the National Museum of Kenya (**NMK**) in Nairobi and the Institute of Zoology, Chinese Academy of Sciences (**IZCAS**) in Beijing, China.

## Systematics

### Family Telemidae Fage, 1913

#### Genus Guhua

Taxon classificationAnimaliaAraneaeTelemidae

Zhao & Li gen. n.

http://zoobank.org/5F7D0378-27F2-47DA-B20A-EAA7449091A7

##### Type species.

*Guhua kakamegaensis* sp. n. from Kakamega County, Kenya.

##### Etymology.

The generic name is taken from the Chinese Pinyin ‘gǔhuà’ meaning sclerotization, referring to the sclerotized receptacle of females. The gender is feminine.

##### Diagnosis.

**Figure 1.**
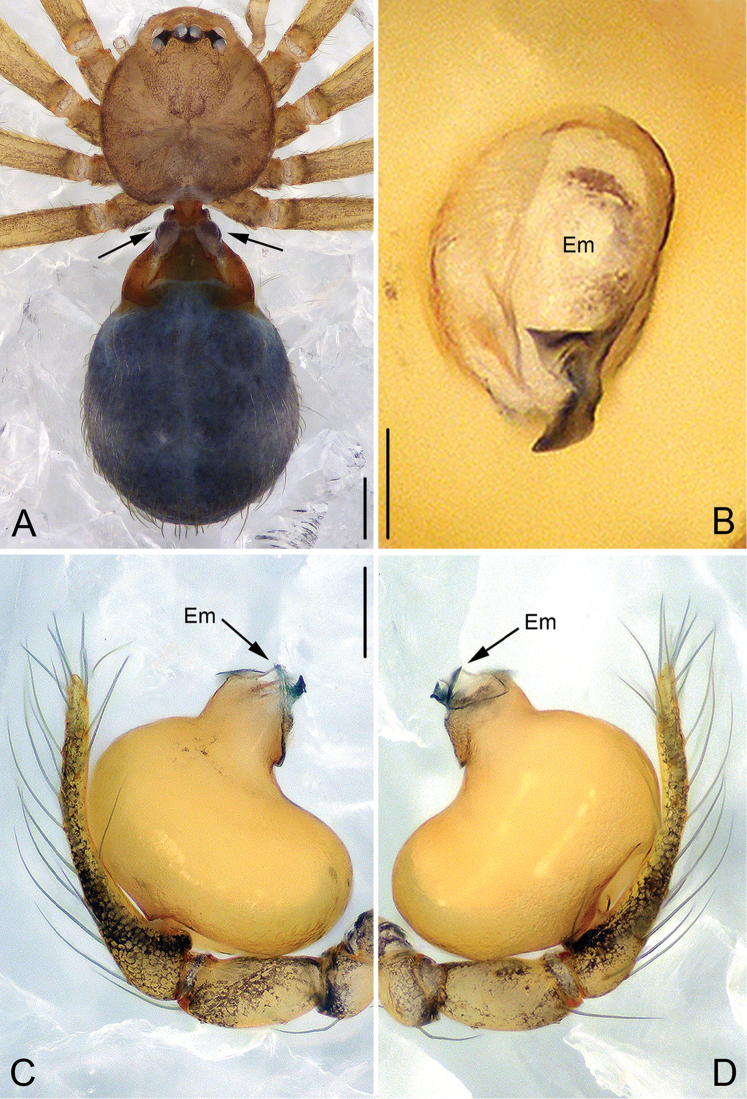
*Guhua kakamegaensis* sp. n., male holotype. **A** Habitus, dorsal view **B** Embolus, apical view **C** Palp, prolateral view **D** Palp, retrolateral view. Scale bars: 0.2 mm (**A**), 0.02 mm (**B**), 0.1 mm (**C, D**). Em, embolus.

**Figure 2.**
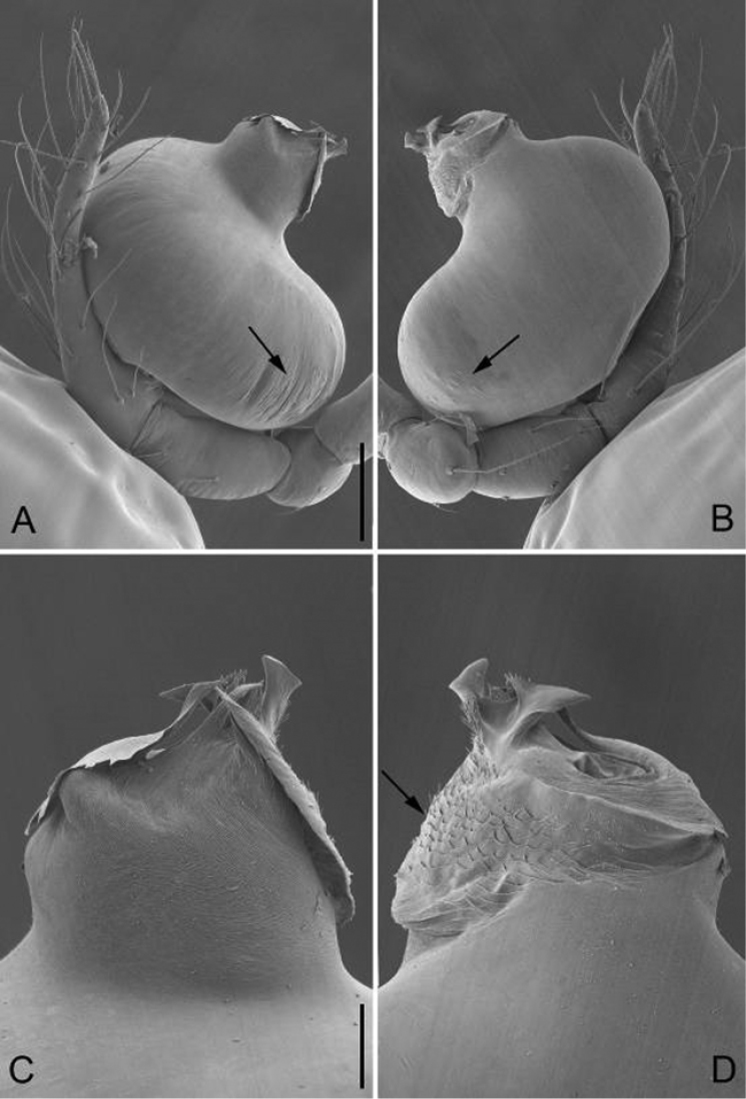
*Guhua kakamegaensis* sp. n., male paratype. **A** Palp, prolateral view **B** Palp, retrolateral view **C** Embolus, prolateral view **D** Embolus, retrolateral view. Scale bars: 0.1 mm (**A, B**), 0.03 mm (**C, D**). Arrows indicate special structures of embolus and bulb.

**Figure 3.**
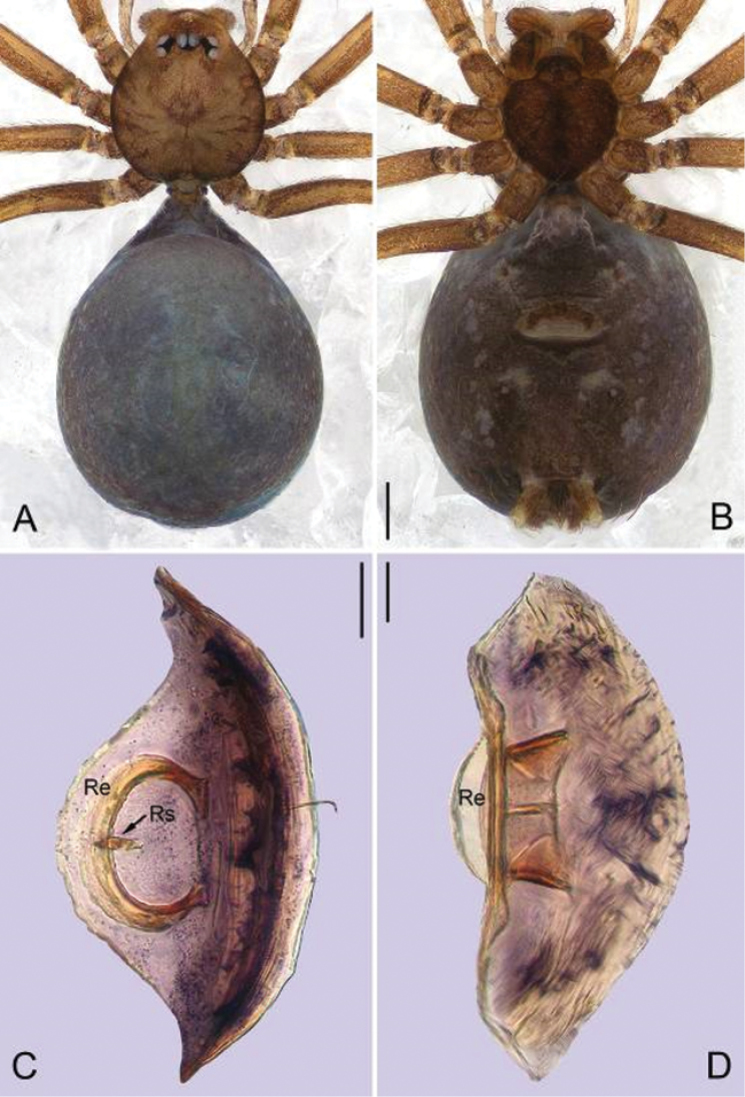
*Guhua kakamegaensis* sp. n., female paratype. **A** Habitus, dorsal view **B** Habitus, ventral view **C** Genitalia, anterior view **D** Genitalia, lateral view. Scale bars: 0.2 mm (**A, B**), 0.05 mm (**C**), 0.02 mm (**D**). Re, receptacle; Rs, receptacle scape.

The new genus can be diagnosed by the following characters: males can be distinguished by an hourglass-shaped lorum (Fig. 1A), extended lateral plates on the anterodorsal surface of the abdomen, two globular apophyses between the lorum and lateral plates (in contrast to a membranous structure in other genera, except *Jocquella leopoldi* Baert, 1980); the male bulb has no tegular apophysis on the middle-upper part of the bulb (Figs 1C–D, 2C–D) (vs. the tegular apophysis or apophyses present in other African genera), no cymbial apophysis on cymbium (Figs 1C, 2A) (vs. a cymbial apophysis in *Pinelema*) or belt-shaped glands on legs (vs. plate-shaped glands in *Telema*); embolus is nearly cylindrical (Figs 1C–D, 2A–B), arising from the anterior surface of the palpal bulb (in most other genera, the embolus is conical, tube-shaped or shaped otherwise with a broad base and rather narrow apex, arising from the apical side of the bulb). Females can be distinguished by the sclerotized and arched receptacle (Fig. 3C–D), as no sclerotized receptacle have been reported in other genera.

##### Description.

See species description.

##### Species composition.

*Guhua kakamegaensis* sp. n.

##### Distribution.

Known only from Kenya.

##### Note.

It is presumed that this new genus was the first record of a sclerotized receptacle in Telemidae. In *Cangoderces lewisi* Harington, 1951, the receptacle was also described as having a relatively sclerotized tube (Brignoli 1978). However, that description is considered to be inaccurate, because the endogyne is similar to most telemids’ receptacle and has usually been described as membranous rather than sclerotized.

#### Guhua kakamegaensis

Taxon classificationAnimaliaAraneaeTelemidae

Zhao & Li sp. n.

http://zoobank.org/A992EFDF-7514-442E-B881-524A79DD0526

Figs 1 , 2 , 3 , 7 , 8 , 10

##### Type material.

**Holotype** ♂ (NMK): Kenya: Kakamega County: Kakamega Town, Kakamega Forest, N00°21.13', E34°52.65', 1542 m, 5.VIII.2016, G.M. Kioko, Q.Y. Zhao & Z.Y. Yao. **Paratypes**: 1♂ and 3♀ (IZCAS), same data as holotype.

##### Etymology.

The specific name refers to the type locality; adjective.

##### Diagnosis.

See genus diagnosis.

##### Description.

**Male (holotype).** Total length 1.68. Carapace 0.65 long, 0.58 wide. Abdomen 1.01 long, 0.70 wide. Carapace yellow, with dark spots in the mid-thoracic area and distinct radial stripes around it (Fig. 1A). Chelicerae and legs yellow, with dark brown pattern. Six eyes, all well-developed with black rings around them, clypeus 0.08 long. Cheliceral promargin with two large teeth and four tiny granulous denticles, retromargin with four triangular denticles. Labium, endites, and sternum dark brown. Leg measurements: I 4.56 (1.28, 0.22, 1.41, 1.01, 0.64); II 3.98 (1.18, 0.21, 1.19, 0.85, 0.55); III 3.01 (0.90, 0.20, 0.84, 0.63, 0.44); IV 3.97 (1.20, 0.17, 1.14, 0.90, 0.56). Two trichobothria and one seta on tibia IV (Fig. 8A). Tibial glands distinct and belt-shaped (Fig. 8B–C), the arrangement of secretory orifices is wave-shaped within a smooth striped tegument (Fig. 8B). Lorum and lateral abdominal plates distinct (Fig. 1A). Abdomen dark green, with dense hairs (Fig. 1A).

*Palp*: tibia 1.8 times longer than patella, cymbium bent and slender and 2.4 times longer than tibia. Bulb kidney shaped, with a few wrinkles and papillae basally (arrowed on Fig. 2A–B). Embolus cylindrical, with a complex membranous apex (Figs 1B–D, 2A–D), its retrolateral surface rough and covered by many thorn-shaped structures (arrowed on Fig. 2D).

**Female.** Total length 1.82. Carapace 0.61 long, 0.56 wide. Abdomen 1.14 long, 0.95 wide. Eyes encircled by black rings, clypeus 0.08 long. Other coloration and pattern same as in male (Fig. 3A–B). Abdomen dark brown, with few hairs. Leg measurements: I 3.92 (1.11, 0.19, 1.21, 0.82, 0.59); II 3.44 (0.98, 0.19, 1.05, 0.71, 0.51); III 2.57 (0.75, 0.17, 0.70, 0.53, 0.42); IV 3.62 (1.09, 0.19, 1.06, 0.77, 0.51). Receptacle arch-shaped, sclerotized, with a sclerotized scape arising inward mesally (Fig. 3C–D).

##### Habitat.

Leaf litter in rainforest.

##### Distribution.

Known only from the type locality (Fig. 10).

#### Genus Apneumonella

Taxon classificationAnimaliaAraneaeTelemidae

Fage, 1921

##### Type species.

*Apneumonella oculata* Fage, 1921: 528, figs II 1–4.

#### Apneumonella taitatavetaensis

Taxon classificationAnimaliaAraneaeTelemidae

Zhao & Li sp. n.

http://zoobank.org/2AC0811D-4EBC-494D-917F-0F3ECCB7221E

**Figure 4.**
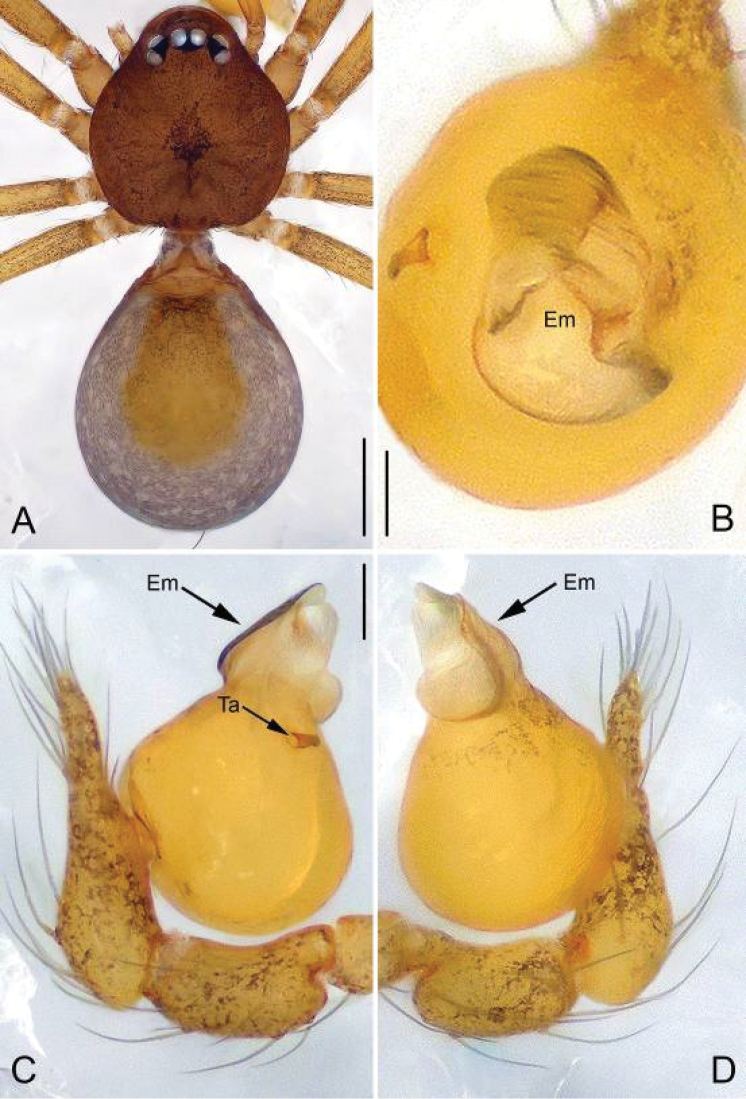
*Apneumonella taitatavetaensis* sp. n., male holotype. **A** Habitus, dorsal view **B** Bulb, apical view **C** Palp, prolateral view **D** Palp, retrolateral view. Scale bars: 0.2 mm (**A**), 0.02 mm (**B**), 0.1 mm (**C, D**). Em, embolus; Ta, Tegular apophysis.

**Figure 5.**
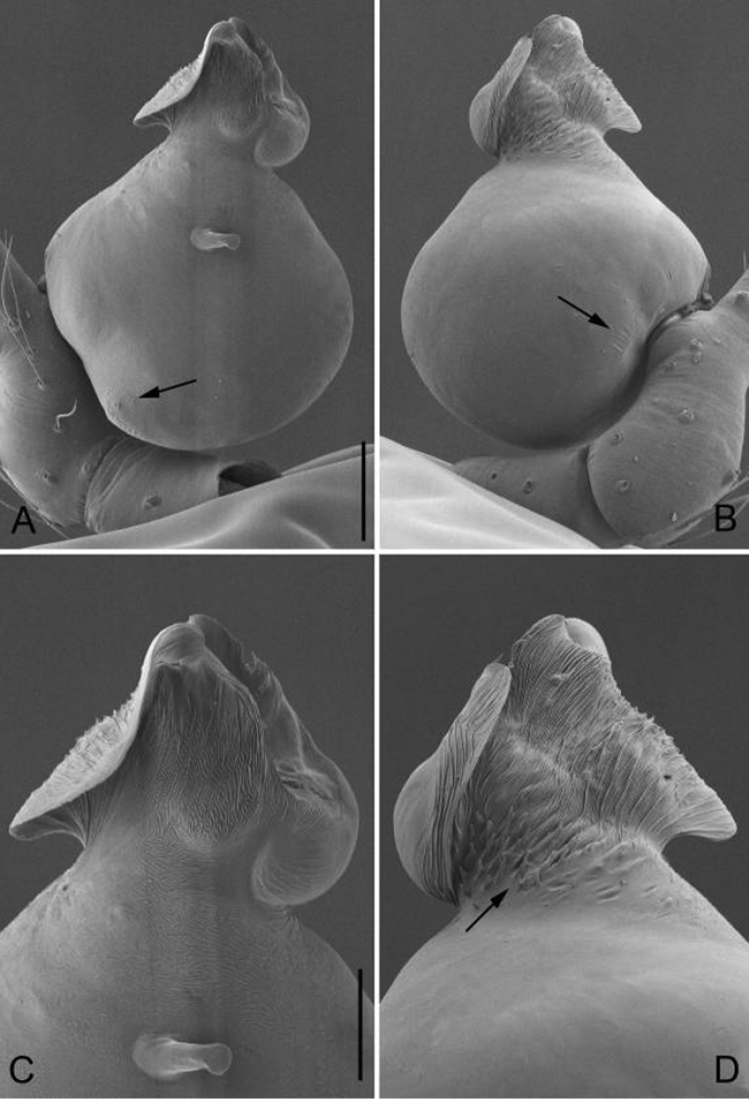
*Apneumonella taitatavetaensis* sp. n., male paratype. **A** Palp, prolateral view **B** Palp, retrolateral view **C** Embolus, prolateral view **D** Embolus, retrolateral view. Scale bars: 0.05 mm (**A, B**), 0.03 mm (**C, D**). Arrows indicate special structures of the embolus and bulb.

**Figure 6.**
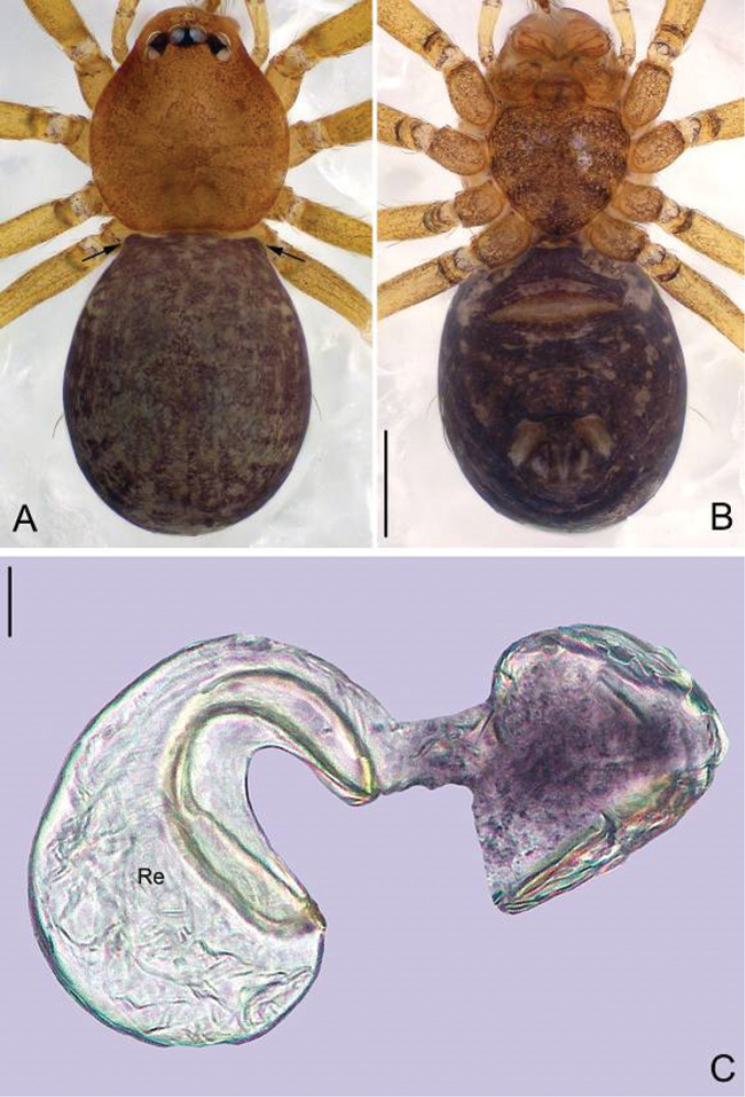
*Apneumonella taitatavetaensis* sp. n., female paratype. **A** Habitus, dorsal view **B** Habitus, ventral view **C** Genitalia, lateral view. Scale bars: 0.2 mm (**A, B**), 0.02 mm (**C**). Re, receptacle. Arrows indicate apophyses of female abdomen.

**Figure 7.**
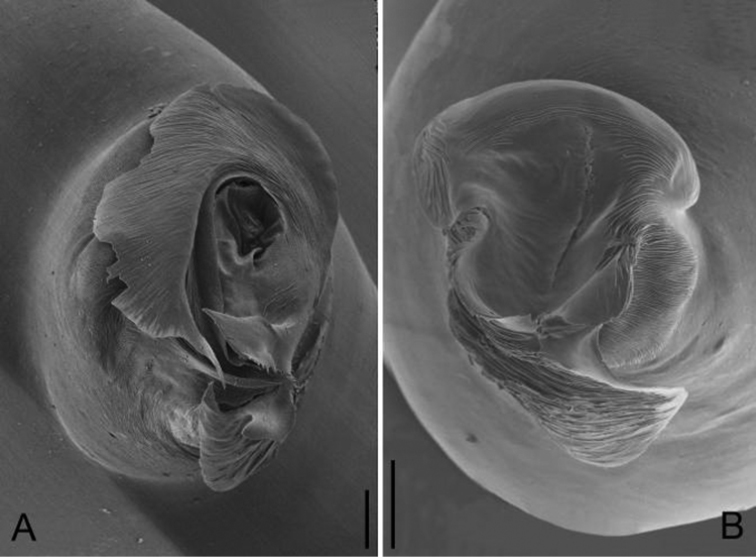
Apical view of embolus. **A** *Guhua kakamegaensis* sp. n. **B** *Apneumonella taitatavetaensis* sp. n. Scale bars: 0.02 mm (**A, B**).

Figs 4 , 5 , 6 , 7 , 9 , 10

##### Type material.

**Holotype** ♂(NMK). Kenya: Taita-Taveta County: Wundanyi Town, Ngangao Forest, S03°21.30', E38°20.41', 1821 m, 23.VII.2016, G.M. Kioko, Q.Y. Zhao & Z.Y. Yao. **Paratypes.** 1♂ and 3♀ (IZCAS), same data as holotype.

##### Etymology.

The specific name refers to the type locality; adjective.

##### Diagnosis.

This new species is similar to *A. oculata* but females can be distinguished by their globular abdomen with two outgrowths near the carapace (arrowed on Fig. 6A), which are not present in *A. oculata*. Another difference is that the diameter of the receptacle is four times the diameter of the insemination duct (Fig. 6C) as compared to that of *A. oculata*, whose receptacle is twice the diameter of the insemination duct. The ocular quadrangle width is half of the carapace width while that of *A. oculata* is one-third of the carapace width.

##### Description.

**Male (holotype).** Total length 1.06. Carapace 0.45 long, 0.41 wide. Abdomen 0.58 long, 0.46 wide. Carapace reddish brown, with dark spots on the mid-thoracic area and obscure radial stripes around it (Fig. 4A). Six eyes, well-developed, encircled by black rings, clypeus 0.05 long. Chelicerae, labium, endites, sternum and legs yellow, with dark brown pattern. Cheliceral promargin with 2 big teeth and 5 granulous denticles and retromargin with 4 triangular denticles. Leg measurements: I 2.10 (0.62, 0.16, 0.59, 0.38, 0.35); II 1.75 (0.53, 0.12, 0.46, 0.34, 0.30); III 1.41 (0.40, 0.10, 0.33, 0.34, 0.24); IV 1.83 (0.54, 0.11, 0.50, 0.38, 0.30). Secretory orifices of tibial glands round (Fig. 9B–C). Abdomen light brown with a yellow spot centrally.

*Palp*: tibia thick, 1.7 times longer than patella; cymbium straight and thick, 1.8 times longer than tibia. Bulb ovoid, with a finger-like tegular apophysis on its middle-upper part and several wrinkles basally (arrowed on Fig. 5A–B). The bulb apex extends into a nearly conical embolus (Figs 4B–D, 5A–D), the surface of the embolus is rough with dense spine-like structures (arrowed on Fig. 5D).

**Female.** Total length 0.98. Carapace 0.42 long, 0.37 wide. Abdomen 0.55 long, 0.46 wide. Eyes encircled by black rings, clypeus 0.04 long. Carapace light brown, sternum dark brown. Abdomen globular with two outgrowths near carapace (arrowed on Fig. 6A). Other coloration and pattern similar to male (Fig. 6A–B). Leg measurements: I 1.83 (0.54, 0.13, 0.50, 0.34, 0.32); II 1.58 (0.48, 0.11, 0.43, 0.28, 0.28); III 1.34 (0.38, 0.11, 0.35, 0.27, 0.23); IV 1.78 (0.53, 0.11, 0.48, 0.39, 0.27). Insemination duct thin and short, receptacle membranous and baglike with a single tube gradually expanding as a concave sac, and the diameter of receptacle four times larger than that of the insemination duct (Fig. 6C).

**Figure 8.**
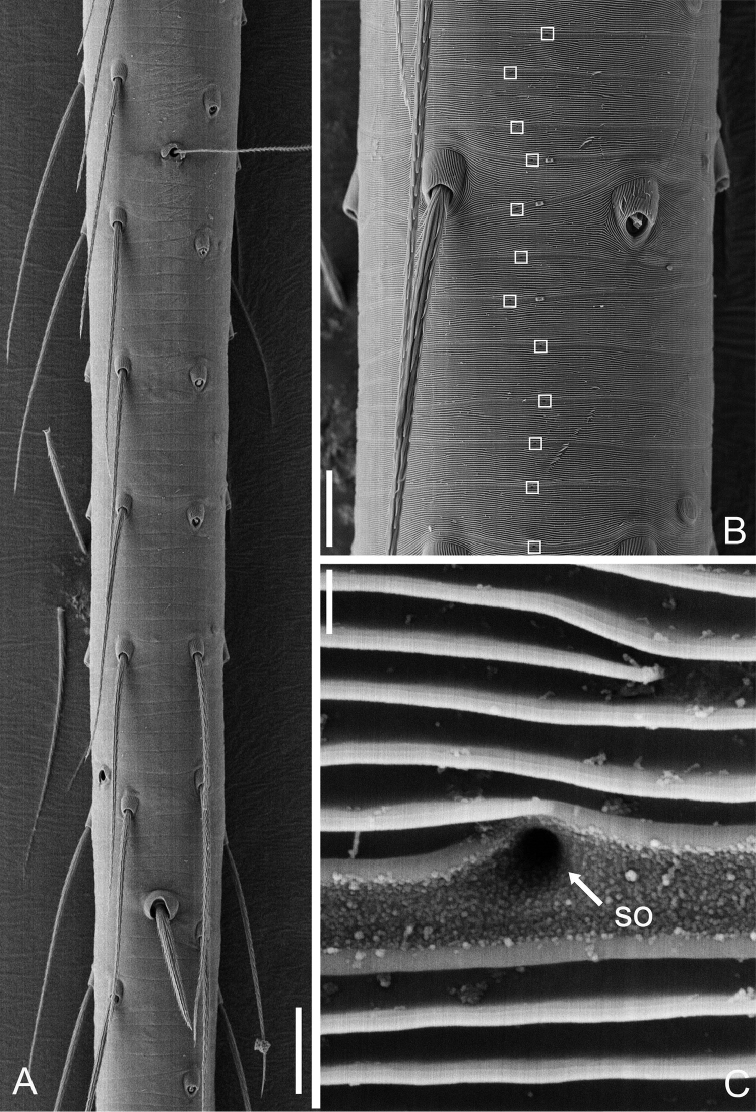
Tibial glands of *Guhua kakamegaensis* sp. n. (**A–C**). **A** Part of tibia **B** Position of tibial glands (white squares) **C** Secretory orifice (SO). Scale bars: 30 μm (**A**), 10 μm (**B**), 0.3 μm (**C**).

**Figure 9.**
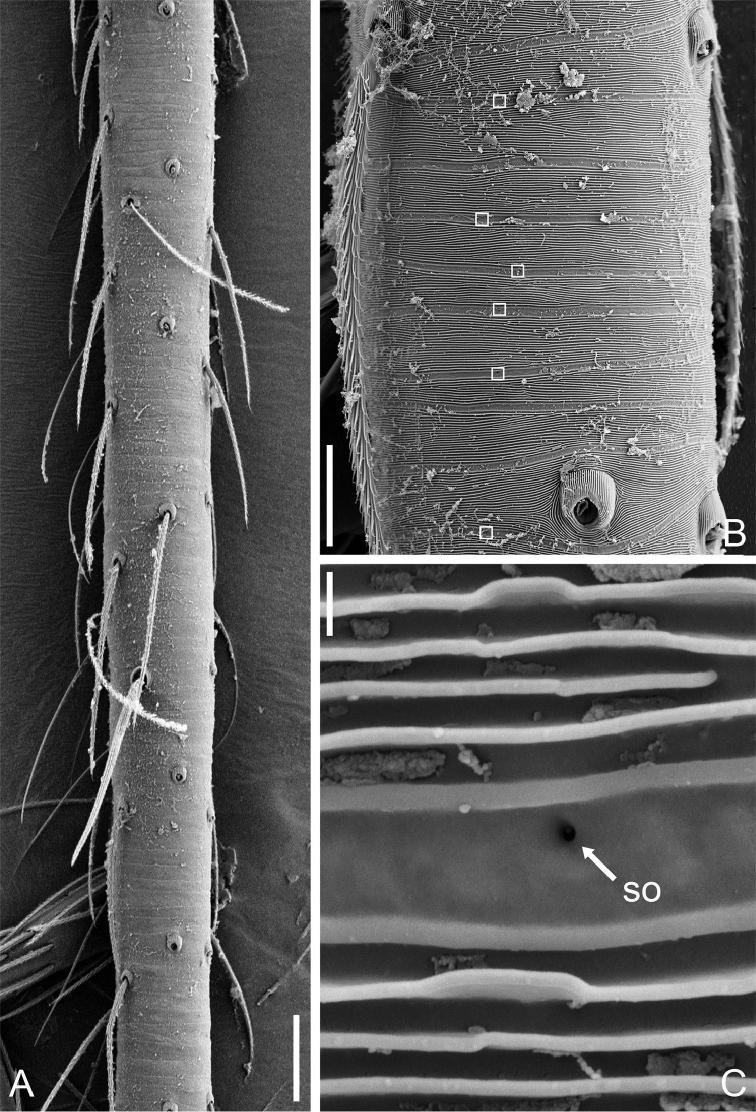
Tibial glands of *Apneumonella taitatavetaensis* sp. n. (**A–C**) **A** Part of tibia **B** Position of tibial glands (white squares) **C** Secretory orifice (SO). Scale bars: 30 μm (**A**), 10 μm (**B**), 0.3 μm (**C**).

**Figure 10.**
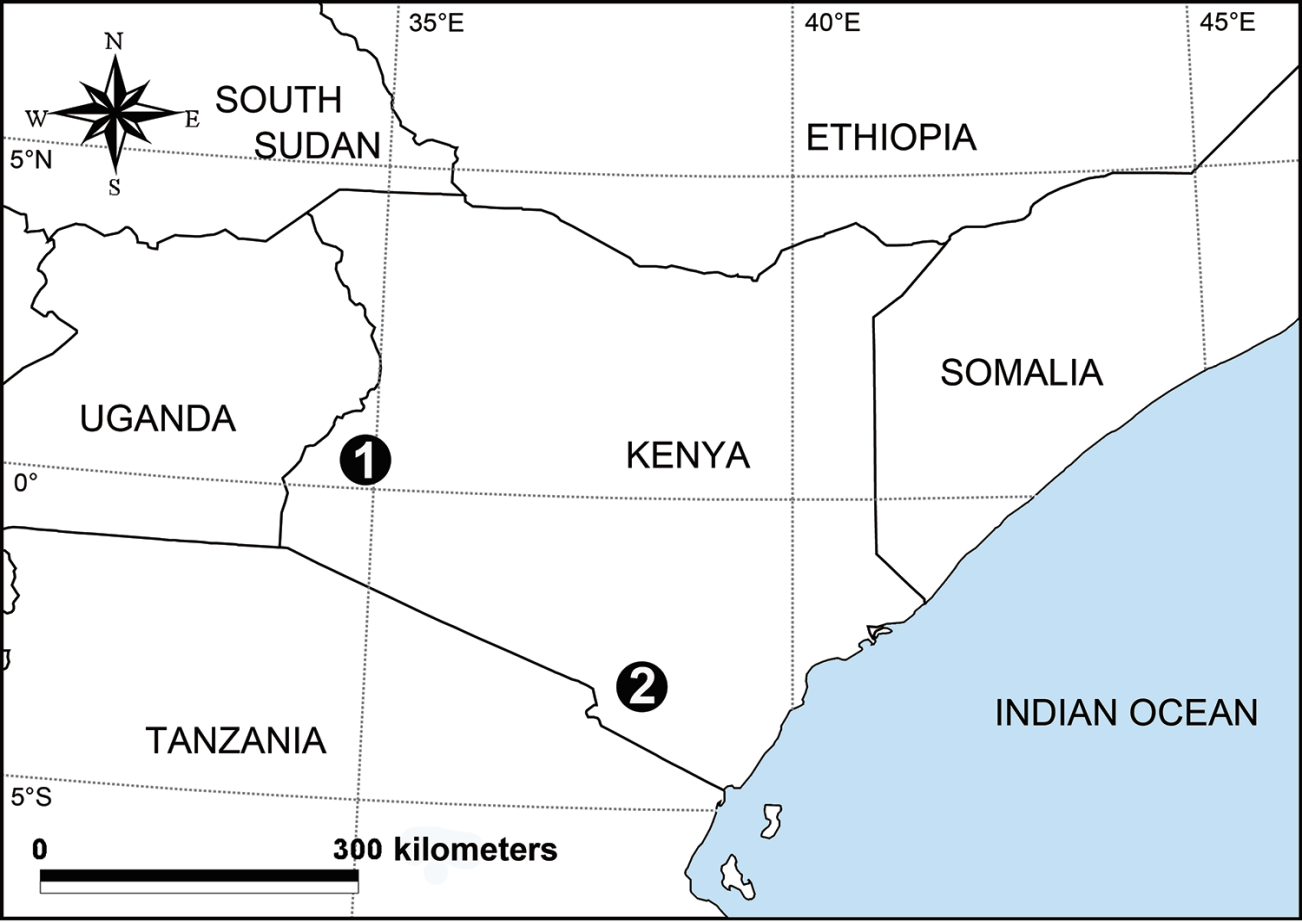
Distribution records of Telemidae in Kenya: **1** *Guhua kakamegaensis* sp. n. **2** *Apneumonella taitatavetaensis* sp. n.

##### Habitat.

Leaf litter in cloud forest.

##### Comments.

*Apneumonella* Fage, 1921 was described with *A. oculata* Fage, 1921 from Tanzania as the type species. The new species shares several characters with *A. oculata*, including the shape of the receptacle and denticulation of the chelicerae. Furthermore, the shape of tibial glands in this species is different from the plate-shaped structure of the tibial glands in *Telema* and *Usofila*, but similar to the lined structure in *Apneumonella* (Emerit 1985). The collection locality of *A. taitatavetaensis* sp. n. is approximately 150 km from the type locality of *A. oculata*.

##### Distribution.

Known only from the type locality (Fig. 10).

## Supplementary Material

XML Treatment for Guhua

XML Treatment for Guhua kakamegaensis

XML Treatment for Apneumonella

XML Treatment for Apneumonella taitatavetaensis
